# Evolutionary dynamics of human autoimmune disease genes and malfunctioned immunological genes

**DOI:** 10.1186/1471-2148-12-10

**Published:** 2012-01-25

**Authors:** Soumita Podder, Tapash Chandra Ghosh

**Affiliations:** 1 Bioinformatics Centre, Bose Institute, P 1/12, C.I.T. Scheme VII M, Kolkata 700 054, India

**Keywords:** Autoimmune disease, Immunological genes, Evolutionary rate, SNPs, Alternative splicing

## Abstract

**Background:**

One of the main issues of molecular evolution is to divulge the principles in dictating the evolutionary rate differences among various gene classes. Immunological genes have received considerable attention in evolutionary biology as candidates for local adaptation and for studying functionally important polymorphisms. The normal structure and function of immunological genes will be distorted when they experience mutations leading to immunological dysfunctions.

**Results:**

Here, we examined the fundamental differences between the genes which on mutation give rise to autoimmune or other immune system related diseases and the immunological genes that do not cause any disease phenotypes. Although the disease genes examined are analogous to non-disease genes in product, expression, function, and pathway affiliation, a statistically significant decrease in evolutionary rate has been found in autoimmune disease genes relative to all other immune related diseases and non-disease genes. Possible ways of accumulation of mutation in the three steps of the central dogma (DNA-mRNA-Protein) have been studied to trace the mutational effects predisposed to disease consequence and acquiring higher selection pressure. Principal Component Analysis and Multivariate Regression Analysis have established the predominant role of single nucleotide polymorphisms in guiding the evolutionary rate of immunological disease and non-disease genes followed by m-RNA abundance, paralogs number, fraction of phosphorylation residue, alternatively spliced exon, protein residue burial and protein disorder.

**Conclusions:**

Our study provides an empirical insight into the etiology of autoimmune disease genes and other immunological diseases. The immediate utility of our study is to help in disease gene identification and may also help in medicinal improvement of immune related disease.

## Background

The knowledge gleaned from several *in silico *studies has facilitated in understanding the variability of evolutionary patterns in gene classes that can illuminate their inherent characteristics. In particular, studies on the functional and evolutionary attributes of human immune system have attained a major focus since it is an orchestra of various defense mechanisms whereby human body maintains functional and organizational integrity against foreign encroachment. The evolutionary history of insects, chicken and mammals indicates that the majority of immune response genes are subjected to positive selection than remainder of the genes [1-3]. Immune response genes are also found to exhibit rapid gene turn over i.e. gene gain and loss [4]. Contextually, it has been proposed that probability of disease predisposition is higher in the genes with high rates of non synonymous mutations [5]. Diseases caused by abnormal or absences of immunologic mechanisms are thus very much common. According to disease mechanism, immune system linked disease genes are generally categorized into two broad classes (i) Immunodeficiency-dysregulation of the immune system in eliminating microbial antigens resulting in chronic immunologic inactivation predisposed to immunologic disorder such as AIDS, DiGeorge syndrome, Chronic granulomatous disease, Wiskott-Aldrich Syndrome, Hypersensitivity etc [6]. (ii) Autoimmunity- mistakenly immune system launches attacks on its own tissue by confusing itself as a foreign invader, leading to autoimmune disorder e.g. Graves disease, Rheumatoid arthritis, Multiple sclerosis, Goodpasture's syndrome etc [6]. Till date various analyses with autoimmune diseases have attempted to figure out their novel characteristics and possible mechanisms [7-10]. Recently, it has been hypothesized that some evolutionarily conserved proteins, present in pathogenic, commensal organisms and their hosts, provide the stimulus that initiates autoimmune disease in susceptible individuals [9]. A possible mechanism of autoantigen formation was thought to be instigated by increased non-canonical splicing that renders untolerized epitopes on antigen [10]. Although disorders caused by dysregulation of immune system have been studied in separate disease class, unique features of that entire disease genes class are still uncharacterized.

Recently, more extensive focus has been concentrated to scrutinize the disease genes for their unique characteristics that distinguish them from the remainder of the genome [11-13]. The growing incidence of autoimmune and several immunological diseases have prompted us to delve into the genic or proteomic features which induce the disease causing mutation on host defense genes. Evaluating the properties of functional immunological genes, malfunctioned immunological genes and autoimmune disease genes in the evolutionary framework we postulate that the autoimmune disease genes are under the strongest purifying selection among the three classes. We exemplified the underlying reasons by assessing the mutational effect at DNA-mRNA-protein levels. The comprehensive cataloguing and characterization of genes from evolutionary perspective may provide the basis for determining how nucleotide substitution impacts biological function and instigate common human diseases. Identification of the various features that are responsible to distinguish between several immunological disease and non-disease genes may help to identify the probable biochemical basis for the disease incidence. Our work may be extended in future in the form of refining the specialized features of functional and disease causing immunological genes.

## Results

### Evolutionary Dynamics of Immune Related Disease and Non-disease Genes

The available resources of immunological disease genes facilitate us to investigate the evolutionary pressure acting on the autoimmune disease genes (AD) and other classes of disease genes resulting from malfunctioning immunological genes (ID) with respect to the immunological genes (IG) without known association to disease. Our result depicts a significant [*P *value = 7 × 10^-3 ^(AD vs ID); 1 × 10^-3 ^(ID vs IG); 4.6 × 10^-2 ^(AD vs IG)] gradual increase in the ratio of non-synonymous to synonymous substitutions (ω) [AD (mean ω = 0.336); ID (mean ω = 0.344); IG (mean ω = 0.446)]. Such evolutionary dynamics of disease and non-disease genes linked to immunological genes is a bit surprising because disease genes intuitively experience more mutational changes than non-disease genes, yet they are unable to escape the evolutionary pressure. It is obvious that mutation or variation will occur either in gene or m-RNA or protein level or in all the three levels to confer disease phenotypes. We intend to investigate in which level the mutation persuade the persistence of selection pressure.

### Effects of Gene level variations

The first wave of information from the human genome analysis has revealed that single nucleotide polymorphisms (SNPs) is the major resource of genetic and phenotypic variations in human. Scanning for the signatures of positive selection in human population suggests that SNPs in protein coding regions show regional evidence of less intense purifying selection [14,15]. Investigating the impact of SNPs in the coding region of above gene classes exemplified that accumulation of non-synonymous SNPs is significantly higher (Z score = 2.37, confidence level = 95%) in case of the AD (78.36%) compared to the ID (73.04%) genes. Moreover, IG genes are themselves less prone to non-synonymous substitutions (69.05%) than both classes of disease genes [AD Vs IG (Z score = 5.0125 confidence level = 95%); ID Vs IG (Z score = 2.011 confidence level = 95%)]. This observation clearly depicts that the most conserved group of genes is indeed the most sensitive ones to variation.

Secondly, the shuffling of genes brought about by genetic recombination is a major engine of genetic variation. Recombination rate (RR) has been found to have a positive correlation with DNA diversity in many organisms, both in animals [16-18] and in plants [19]. Thus, accumulation of higher amount of SNPs was expected to initiate the higher RR for AD compared to ID and IG and the result was also in accordance to the expectation (average RR (cM/Mb) for AD = 0.051, ID = 0.035, IG = 0.0023; each value is significant at least at *P*<0.05 level in Mann-Whitney test). Though the mutagenic nature of recombination rate may reflect the possibility of higher non-synonymous substitutions, the prevalence of Hill-Robertson interference in the genomic regions with higher RR have been proposed to increase the efficacy of purifying selection [20,21]. Moreover, a positive association has been asserted between RR and gene expression level which also explains the lower evolutionary rate in regions with higher recombination frequencies [22]. Analyzing microarray expression data we also observed that on average the AD genes tend to be more highly expressed than the other two classes of genes (average expression for AD = 238.266; ID = 175.138; IG = 128.497; each value is significant at least at *P*<0.05 level in Mann-Whitney test). In addition to that, RR has long been thought to be one of the principal forces behind the gene duplication frequency [23,24]. Calculating the paralogs number in three groups of genes emphasized that the AD genes acquired a large number of duplicates compared to ID and IG genes (Average paralogs per genes for AD = 10.006; ID = 8.61, IG = 6.32; each value was significant at least at *P*<0.05 level in Mann-Whitney test). Higher duplicability may enforce the slower evolutionary rate on AD genes in contrast to other two classes since duplicated genes encounter more purifying selection than singletons even though shortly after the duplication, they experience a considerable relaxation of selection pressure [25]. From this it can be inferred that the SNPs and recombination rate collectively incite recurrent gene duplication (Spearman's ρ _SNP, RR _= 0.120, P = 1.0 × 10^-3^; Spearman's ρ _RR, paralogs number _= 0.060, P = 1.0 × 10^-3^) and elicit the selection pressure on disease genes.

With the advent of genome scanning technology it has uncovered that the human genome becomes structurally dynamic due to the presence of thousands of heritable copy of mutation and are equally important as SNPs [26]. It was reported that reduced purifying selection has been acting upon copy number variants (CNVs) region [27]. Looking for the association of CNVs with immunological disease and non-disease genes we noticed that the non-disease immunological genes are significantly (Z-value = 1.96 at 95% confidence level) more prone (53.98% of total immunological genes) to suffer from CNVs compared to other immunological disease genes (49.72% of total other immunological disease genes) while the later group of disease genes (44.5% of total autoimmune disease genes) exhibit significantly (Z value = 1.99 at 95% confidence level) lesser CNVs than other immunological disease genes. These findings are also consistent with the notion that the CNV genes prefer to encode large numbers of secreted, olfactory, and immunity proteins rather than the genes harboring Mendelian disease [28]. Although the disease genes concerned in our study are inherited by both Mendelian and non-Mendelian fashion, we did not observe any opposite trend for accounting the non-Mendelian disease genes.

### Effects of Transcript level Variations

Over the past decade, it has been postulated that alternative splicing (AS) is a critical post transcriptional event directing an enhancement of transcriptome and proteome diversity, particularly in higher organisms [29]. The frequent accumulation of non-synonymous mutations in alternatively spliced regions [30] initiates a faster rate of evolution in alternatively spliced exons than the constitutively spliced ones as evidenced from a comparison of orthologous human and mouse genes [31]. Investigation on the involvement of the three groups of genes in alternative splicing mechanism revealed that most of the IG genes favor alternative splicing to increase their proteomic diversity in contrast to AD and ID genes (Table 1). Accordingly, the profuse number of alternatively spliced exons are encompassed in IG genes compared to ID and AD genes (average alternatively spliced exons per gene in AD = 5.89, ID = 6.78, IG = 8.85; each value is significant at least at *P*<0.05 level in Mann-Whitney test). Such nature of IG is also biologically relevant since it was proposed that AS is crucial for a functional immune system as it offers the potency of high degree of diversity and the competence of individual cells to rapidly adapt and respond towards the changing environmental conditions [32,33].

**Table 1:** Propensity of three classes of genes involved in different Alternative Splicing associated processes and their *Z - *values of pair wise comparisons.

	Propensity of genes (%)				Propensity of genes (%)				Propensity of genes (%)		
	AD	ID	IG		AD	ID	IG		AD	ID	IG
**Alternative Splicing**	79.5	84.38	89.79	**NMD-linked mRNA decay**	11.65	18.2	34	**5'splice Site SNPs**	23.77	14.58	8.73
**Significant Level (at 95% confidence level)**	*Z *= 2.6 (AD vs. ID)	*Z *= 3.9 (ID vs. IG)	*Z *= 7.5 (IG vs. AD)		*Z *= 1.7 (AD vs. ID)	*Z *= 5.5 (ID vs. IG)	*Z *= 7.6 (IG vs. AD)		*Z *= 1.03 (AD vs. ID)	*Z *= 4.8 (ID vs. IG)	*Z *= 7.5 (IG vs. AD)

Since, alternative splicing can bolster organism complexity by effectively increasing the proteome size, the m-RNA abundance would be higher for the immunological genes. However, we already noticed IG genes are lowly expressed. Accounting EST data, the trend remain exactly same i.e. the EST count/m-RNA abundance is lower for the IG (27.02) compared to ID (35.11) and AD (48.72) genes. Hence, we ask what drives the lower m-RNA abundance of AS rich immunological genes. In the recent year it has been clarified that up to one-third of human AS events create a premature termination codon (PTC) that would cause the resulting mRNA to be degraded by nonsense-mediated mRNA decay (NMD) [34,35] and it was also stated that a higher rate of mRNA decay can be considered as an indicator of the lower gene expressivity [36]. Analysis on the coupling of NMD to the AS linked genes shows that most of the alternatively spliced isoforms of IG undergo mRNA decay while the count is much lower for ID and AD genes (Table 1).

Another implication of alternative splicing is to promote intrinsically disordered protein, thus enabling functional and regulatory diversity in human proteome [37,38]. Calculation of disorder residues in the three classes of proteins shows that the percentage of unstructured protein regions in IG, ID and AD genes are respectively 44.23%, 32.22% and 21.52% and the difference between each of the above values is significant at P < 0.05 (in Mann-Whitney test). The aberrant increase of disorderness in IG proteins again confirms the high flexibility of antigen binding sites in immunoglobulin

to combat against an almost infinite diversity of physiological or synthetic antigens is predominantly rendered by intrinsically disordered regions of proteins [39]. Association with a large number of disorder residues of IG is also be an imperative reason for their faster evolutionary rate than AD and ID genes since in some protein families it has been demonstrated that the disordered regions evolve at a significantly faster rate than the ordered regions [40].

### Role of SNPs on Transcript level Variations

In recent years there has been growing evidence for extensive natural variations like SNPs to be the major contributor of alternative splicing variation in humans [41]. Numerous disease-causing mutations within the consensus 5' splice site create a cryptic splice site that leads to defective mRNA and protein products [42,43]. In our study, we also noticed a greater association of 5' splice site SNPs (ss SNP) with AD genes compared to ID and IG (Table 1). This phenomenon indicates that SNPs impede the disease genes (AD, ID) to take part in alternative splicing by altering the splicing signals and their lower involvement with alternative splicing than IG genes may imposes much more evolutionary pressure on disease genes.

### Effects of Protein level Variations

All proteins are potentially subjected to Post-translational Modifications (PMs) to accomplish many important roles in regulating the biological processes such as regulation of gene expression, activation/deactivation of enzymatic activity, protein stability or destruction, mediation of protein-protein interactions etc [44]. However, in some cases, PMs may be detrimental to protein functionality and may compromise the cellular functions in which they reside [45]. Among many of the modifications, post-translational phosphorylation is one of the most common protein modifications that occur in animal cells. Calculation of PMs sites revealed that the fraction of potential phosphorylation residues i.e serine, threonine, and cysteine to the total length of the protein is significantly (Mann-Whitney's P < 0.05 in each case) higher in case of AD genes (0.097) compared to ID (0.084) and IG (0.069) genes. This observation again emphasized the previous hypothesis that the abnormal frequency of PMs uncover cryptic epitopes or create some novel epitopes that may be not tolerated during T-cell selection and trigger the pathogenesis of autoimmune disorder [45]. Contextually, it has recently been discovered that an additional purifying selection are operated on the positions involved in phosphorylation as compared to their unmodified counterparts in the same protein [46]. Thus the higher enrichment of post-translational phosphorylation site in AD genes may be considered as a potential reason for their lower evolutionary rate.

**Figure 1:**
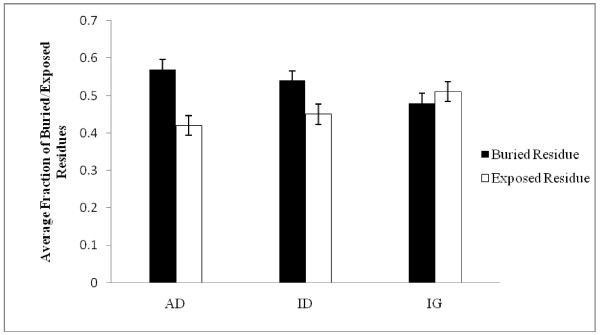
**Distributions of buried and exposed residues among AD, ID and IG proteins**. Error bar represents 5% standard error of data.

Furthermore, it is well established that buried residues in a protein are important determinants of protein stability while surface residues are involved in protein function [47]. Here we found that AD genes bury more residue on average compared to ID and IG genes (Figure 1). Since buried residues evolve at a slower rate [48], the higher level of residue burial in AD genes can be accounted for their lower sequence divergence and as well as a possible means of achieving greater stability.

### Role of SNPs on Protein level Variations

**Figure 2:**
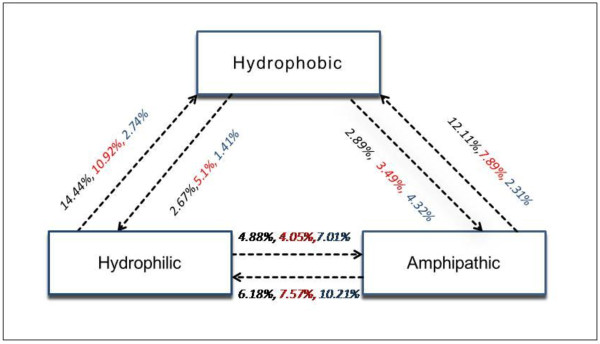
**Average amino acid exchange frequencies due to Single Nucleotide Polymorphisms (SNPs) among hydrophobic, amphipathic and hydrophilic amino acids for AD (black), ID (red), IG (blue) proteins**.

**Figure 3:**
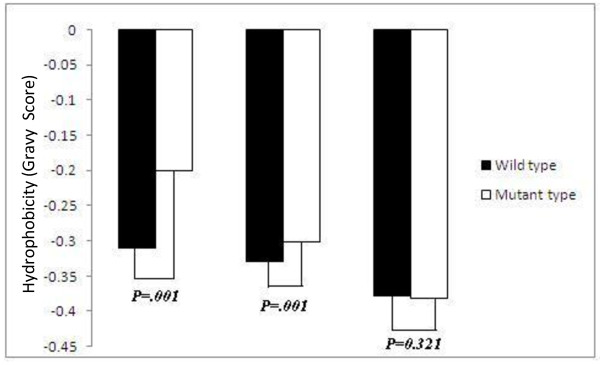
**Differences in average hydrophobicity score between the three categories of genes before mutation (wild type) and after mutation (mutant type) with SNPs**. P-value shows the significant level.

Systematic approach to the analysis of SNPs indicated that SNPs resulting in deleterious amino acid changes predominantly affect the stability of the protein [49]. We then map the non-synonymous SNPs on protein buried region and quantify the hydrophobic, hydrophilic, amphipathic amino acid substitution frequency in each group of genes. The average amino acid exchange frequencies among hydrophobic, hydrophilic and amphipathic amino acids among AD, ID, IG genes for buried regions of proteins are diagrammatically represented in Figure 2. We noticed transition from hydrophilic to hydrophobic or amphipathic to hydrophobic residue is more frequently substituted in the buried regions of AD proteins compared to ID and IG proteins. Moreover, the hydrophobicity of buried region in AD genes has found to increase significantly after substitution with SNPs than ID genes while no change of hydrophobicity has occurred in case of IG (Figure 3). Thus, influence of SNPs in increasing the hydrophobicity in buried region of AD proteins may be responsible for evolutionary constraint for maintaining protein stability.

### Relative Contribution of the Factors in Determining Evolutionary Rate Variation

Here, we noticed different probable factors in the three levels (DNA-m-RNA-Protein) that can explain evolutionary rate differences among AD, ID and IG genes. To assess the contribution of each variable, we compute Principal Component Analysis (PCA). The dominant eigen vectors (taken as equal to or greater than 1) that appear from this analysis can be interpreted as the most important contributors directing protein evolution [50]. PCA with gene level variables (SNPs, CNVs, RR, duplicability); m-RNA level variables (isoform number, alternatively spliced exon, m-RNA abundance, disorderness); protein level variables (phosphorylation, protein residue burial), which are the dominant factors, are represented in table 2. Multiple Regression Analysis was then performed to assess the contribution of each level variables determined in PCA in a single regression model from which we can identify the influence of all potential predictor variables and at the same time can eliminate step by step those predictors that contribute least to the regression model. Regression analysis exhaustively confirmed that the SNPs (β= -3.725), is the most influential predictor of the evolutionary rate followed by the m-RNA abundance (β= -3.005), paralogs number (β= -2.036), fraction of phosphorylation residue (β = -2.091), alternatively spliced exon (β = 1.960), protein residue burial (β= -1.085) and protein disorder (β = 1.021).

**Table 2:** Principal Component Analysis on Evolutionary Rate (ω) with (**a) **Gene Level Predictors, **(b) **Transcript Level Predictors, **(c) **Protein Level Predictors.

a. **Gene Level Predictors**		
	Principal Component1	Principal Component2
**Percent of the total variance** **Correlation Coefficient (Spearman's ρ) with ω**	27.502 -0.061**	21.912 0.113**
**Major Contributing Factor in PCA1**		
**Paralogs Number** **Recombination Rate**	0.789 0.805	--- ---
**Major Contributing Factor in PCA2**		
**Single Nucleotide Polymorphism** **Copy Number Variation**	--- ---	0.995 0.713
a. **Transcript Level Predictors**		
**Percent of the total variance** **Correlation Coefficient (Spearman's ρ) with ω**	39.071 -0.161**	
**Major Contributing Factor in PCA1**		
**m-RNA abundance** **Alternatively Spliced Exons** **Disorderness**	0.702 0 0.688 0.446	
c.**Protein Level Predictors**		
**Percent of the total variance** **Correlation Coefficient (Spearman's ρ) with ω**	69.095 -0.321***	
**Major Contributing Factor in PCA1**		
**Fraction of Phosphorylation Residues** **Proportion of Buried Residues**	0.557 0.743	

N.B.: * denotes *P *< 10^-3^; ** denotes *P *< 10^-6^; *** denotes *P *< 10^-9^.

## Discussion

Recent years have witnessed rapid progress in elucidating the molecular causes of various diseases. Here we analyzed the evolutionary disparity between the functional and non-functional immune systems. We noticed that autoimmune disease genes are more conserved than other immunological disease genes and both sets of genes evolved significantly at a slower rate than immunological genes. Though the evolutionary rates differences among the gene groups are statistically significant, the difference of mean values between autoimmune and immunological disease genes is small. However, the differences of mean values among the groups turned out to be prominent when we analyzed non-synonymous and synonymous substitution rates separately (dn for autoimmune disease genes = 0.0079; immunological disease genes = 0.0091; immunological genes = 0.0118; Mann-Whitney's P = 1 × 10^-3 ^in each case and ds for autoimmune disease genes = 0.0232; immunological disease genes = 0.0254; immunological genes = 0.0291; Mann-Whitney's P < 0.05 in each case). Significant differences in synonymous substitutions rates among the gene groups indicate the role of neutral substitutions in driving the evolutionary rate discrepancies among them. Now, the slower evolving disease linked immune genes raise a fundamental question why non disease immune genes evolve at a higher rate compared to disease related immune genes since it was previously documented by several studies that non-disease genes evolve at a slower rate than disease genes [51,52], though some controversial reports [11] are also present. To resolve this controversy, our previous study [12] exemplified that, monogenic diseases inherited by Mendelian fashion and polygenic disease genes inherited by non-Mendelian fashion are evolutionarily faster than housekeeping genes but monogenic disease genes show slower evolutionary rate than tissue specific genes. It is also noteworthy to mention that immune system genes show tissue-specific expression pattern [53] and both of our disease datasets mostly comprise monogenic disease genes (autoimmune disease genes dataset: 69% monogenic disorder, 31% polygenic disorder; other immunological disease genes dataset:61% monogenic disorder; 39% polygenic disorder). Herein, the differences in single nucleotide polymorphisms, copy number variations, recombination rate, duplicability, alternative splicing, disorderness, post-translational modification, and protein residue burial can explain the evolutionary disparity among the three groups of genes.

**Figure 4:**
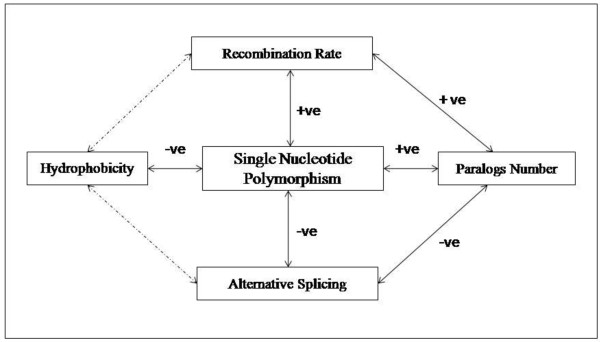
**The schematic representation to illustrate synchronous effect of Single Nucleotide Polymorphisms on recombination rate, hydrophobicity, paralogs number, alternative splicing**. Bold arrows denote significant correlations, +ve for positive and -ve for negative while the dotted arrow indicates non-significant (NS) relationship between variables.

The evolutionary conservation of disease related immunological genes in spite of their higher association with non-synonymous single nucleotide polymorphisms is an artifact of its beneficial impact on disease related genes (Figure 4). Single nucleotide polymorphisms up-regulate recombination rates which in turns increase the gene expression as well as paralogs number in disease genes. Duplication driven disease gene formation has also supported by a series of evidence in an earlier literature [54]. Previously, it was underscored that duplication and alternative splicing could not be operated simultaneously rather they hold a negative correlation with each other [55]. Since the disease genes achieved their proteome size through gene duplication, we observed a lower involvement with alternative splicing. Here also single nucleotide polymorphisms played a critical role in 5' splice site and create a cryptic splice site by altering the splicing signal. On the other hand the immunological genes follow the path of alternative splicing to enhance their diversity. However, the frequent link with alternative splicing could not generate higher m-RNA abundance of immunological genes due to "Regulated Unproductive Splicing and Translation" (RUST) mechanism [56] in which premature termination codon containing isoforms are targeted to non-sense mediated decay to regulate the transcript level of functional protein. Rather alternative splicing helps to impose a greater flexibility to bind with an enormous number of foreign particles without known structural analogy through increasing protein disorderness in immunological genes (Spearman's ρ _disorderness, alternatively spliced exon_= 0.134, P = 1 × 10^-3^). Thus, we deciphered that the basic difference in the involvement of proteome expansion machinery put differential selective pressures on malfunctioning immune genes and the functional immune genes. Moreover, it is also observed in our search that autoimmune disease and other immunological disease genes are more prone to post-translational phosphorylation which may regarded as a possible reason for slower evolutionary rate. In protein structure level, the higher residue burial is observed in two types of disease genes compared to non-disease genes and the propensity of single nucleotide polymorphisms to substitute hydrophilic, amphipathic amino acid by hydrophobic amino acid in disease groups could be prompted as a reason of lower sequence divergence in autoimmune disease and other immunological disease genes than immunological genes. Conferring structural stability to the autoimmune disease genes also has a biological significance since incidence of autoimmunity sharply increases in the stable protein forms in the cell [57].

## Conclusions

Assessing the results from multivariate regression analysis we conclude that the relative dominance of individual factors modulating the differential substitution rate experienced by autoimmune disease, other immunological disease and immunological disease genes is in the order of single nucleotide polymorphisms > m-RNA abundance > paralogs number > phosphorylation residue > alternatively spliced exon > protein residue burial> protein disorder. To the best of our knowledge, this is the first extensive comparison of disease and non-disease related immunological genes from evolutionary perspective. This finding also shades light into the mutational spectrum acting on DNA-mRNA-protein level of the three classes of genes. Our study will surely enrich the knowledge of disease gene identification and may also help in medicinal improvement of autoimmune disease.

## Methods

### Immune Related Disease and Non-disease Genes Identification

Immune related disease genes mainly consist with Autoimmune disease, Immunoproliferative disease, Immunologic deficiency syndromes, hypersensitivity, Graft rejection, Purpura, thrombocytopenia, and Glomerulonephritis. There exists a clear demarcation between the basic disease mechanism of autoimmune disease and rest of the immune related disease genes. Thus immune related disease genes are broadly categorized into the two groups - autoimmune disease genes and other immunological disease genes. These two types of genes inherited by Mendelian and non-Mendelian fashion were downloaded from Biobase and Genetic Association Database [58] respectively. Autoimmune disease genes include Rheumatoid Arthritis, Diabetes Mellitus, Systemic Lupus Erythematosus, Greves disease, Thyroiditis, Antiphospholipid Syndrom, Pemphigus, Polyendocrynopathis, Hemolytic anemia, Multiple Sclerosis etc. Then we have checked the functional description of the gene sets downloaded from Biobase and Genetic Association database. The link between the functional description and disease association was manually verified and the genes whose functional descriptions match with disease associations were considered in our study while the genes which are common in both autoimmune disease and other immunological diseases were excluded from our dataset. Since the main objective of our study is to find out the evolutionary disparity among the gene sets, we have chosen only those genes for which the information is available for their orthologs in Chimpanzee and their dn and ds values in Ensembl. Finally we have constructed the dataset with a total of 781 autoimmune disease genes and 679 other immunological disease genes (Additional file 1, Table S1). Immunological genes were obtained from ImmPort [59] and filtered with similar criteria. Finally we have acquired 2470 non-disease immunological genes by excluding the above disease genes list (Additional file 1, Table S1).

### Orthologs and Paralogs Identification

The gene sequences, paralogs information, pair-wise non-synonymous substitution rates (dn) and synonymous substitution rates (ds) with Chimp (1:1) orthologs corresponding to both types of immunological disease genes as well as non-disease genes were retrieved from Ensembl [60].

### Gene Expression Profile

The gene expression profile data was extracted from BioGPS dataset [61]. The signal intensities across 79 tissues were averaged and were considered as expression level for each gene represented by their corresponding probe id. mRNA abundance of the genes in our dataset was calculated using EST data obtained from DFCI Gene Indices. Gene expression level was estimated by calculating the number of occurrences of each gene among EST sequences from 179 cDNA libraries sampled with at least 10,000 ESTs [62]. Eliminating pathogenic and cancerous libraries, 41 libraries were kept and alignments were made between the coding sequences of the gene groups with the EST dataset using BLASTN program with a sequence matching criterion of 60% identity and 80% overlaps. The overall EST counts for each gene across 41 EST libraries represented their mRNA abundance.

### Measurement of SNPs, CNVs, Recombination Rate

Non-synonymous SNPs and CNVs information were downloaded from Polydoms [63] and Database of Genomic Variants [64] respectively. Chromosome wise gene recombination rates were downloaded from Hapmap project [65]. The recombination rates of the progenitor genes were calculated using the formula ∑ρ_i _**/**l, where ρ_i _stands for recombination rate at a base position and l for the genic length corresponding to that gene [66].

### Alternative Splicing and SNPs Effect

Data for alternatively spliced isoforms and exons for the genes in the dataset were downloaded from the Alternative Splicing Annotation Project [67]. Splice site SNPs information were collected from ssSNP Target [68]. Data for alternatively spliced isoforms that are coupled to mRNA degradation were fetched from AS-ALPS [69].

### Prediction of Intrinsically Disorder Region, Hydrophobicity and Post-translational Phosphorylation

Disorder predictions were carried out using the program FoldIndex [70] implementing the prediction method of Uversky et al. [71]. Post translational phosphorylation in the disease and non-disease related immunological proteins were measured from NetPhos (2.0) [72]. Hydrophobicity values of proteins were retrieved from ProtParam [73]

### Calculation of Buried and Exposed Residues in Protein

Residue-wise burial in proteins was computed by a standalone version of sequence-based prediction program RVP-Net [74]. This program relies on a neural network trained to estimate solvent accessibility of each residue from sequence features and was trained over non-redundant set of protein structures. Predicted relative solvent accessible surface area was converted to a two burial classes (buried/exposed) at 16% cutoff, which roughly corresponds to the median of solvent accessibility distribution in training proteins. The classification of amino acids as hydrophobic, hydrophilic and amphipathic were done according to ref [75].

### Statistical Test

We performed Mann-Whitney U test for pair-wise comparisons since the values are not normally distributed in our dataset. Multiple regression analysis, Principal component analysis were performed for relative contribution analysis of each factors to evolutionary rate. All the statistical tests were carried out by the SPSS (13.0) package.

## Abbreviations

AD: Autoimmune Disease; ID: Immunological Disease; IG: Immunological Genes; RR: Recombination Rate; AS: Alternative Splicing; NMD: Non-sense Mediated mRNA Decay; PMs: Posttranslational Modifications; ssSNP: splice site SNPs; PCA: Principal Component Analysis; PTC: Premature Termination Codon.

## Competing interests

The authors declare that they have no competing interests.

## Authors' contributions

SP made the analyses and drafted the manuscript. TCG guided the work and helped to draft the manuscript. All authors read and approved the final manuscript.

## Supplementary Material

Additional file 1 **Supplementary Table S1**. Genomic and proteomic features of Autoimmune disease, Immunological disease gens and Immunological genes. Click here for file

## References

[B1] Hughes AL Packer B Welch R Chanock SJ Yeager M High level of functional polymorphism indicates a unique role of natural selection at human immune system loci Immunogenetics 2005 57 821 827 10.1007/s00251-005-0052-7 16261383

[B2] Park SG Choi SS Expression breadth and expression abundance behave differently in correlations with evolutionary rates BMC Evol Biol 2010 10 241 10.1186/1471-2148-10-241 20691101 PMC2924872

[B3] Limaye N Belobrajdic KA Wandstrat AE Bonhomme F Edwards SV Wakeland EK Prevalence and evolutionary origins of autoimmune susceptibility alleles in natural mouse populations Genes Immun 2008 9 61 68 10.1038/sj.gene.6364446 18094711

[B4] Hahn MW Demuth JP Han SG Accelerated rate of gene gain and loss in primates Genetics 2007 177 1941 1949 10.1534/genetics.107.080077 17947411 PMC2147951

[B5] Bustamante CD Fledel-Alon A Williamson S Nielsen R Hubisz MT Glanowski S Tanenbaum DM White TJ Sninsky JJ Hernandez RD Natural selection on protein-coding genes in the human genome Nature 2005 437 1153 1157 10.1038/nature04240 16237444

[B6] Thomas JK Richard AG Barbara AO Janis K Kuby Immunology 2006 6 W.h.freeman & Co Ltd

[B7] Henderson RD Bain CJ Pender MP The occurrence of autoimmune diseases in patients with multiple sclerosis and their families J Clin Neurosci 2000 7 434 437 10.1054/jocn.2000.0693 10942666

[B8] Aune TM Parker JS Maas K Liu Z Olsen NJ Moore JH Co-localization of differentially expressed genes and shared susceptibility loci in human autoimmunity Genet Epidemiol 2004 27 162 172 10.1002/gepi.20013 15305332

[B9] Wegner N Wait R Venables PJ Evolutionarily conserved antigens in autoimmune disease: implications for an infective aetiology Int J Biochem Cell Biol 2009 41 390 397 10.1016/j.biocel.2008.09.012 18926919

[B10] Ng B Yang F Huston DP Yan Y Yang Y Xiong Z Peterson LE Wang H Yang XF Increased noncanonical splicing of autoantigen transcripts provides the structural basis for expression of untolerized epitopes J Allergy Clin Immunol 2004 114 1463 1470 10.1016/j.jaci.2004.09.006 15577853 PMC3902068

[B11] Huang H Winter EE Wang H Weinstock KG Xing H Goodstadt L Stenson PD Cooper DN Smith D Albà MM Evolutionary conservation and selection of human disease gene orthologs in the rat and mouse genomes Genome Biol 2004 5 R47 10.1186/gb-2004-5-7-r47 15239832 PMC463309

[B12] Podder S Ghosh TC Exploring the differences in evolutionary rates between monogenic and polygenic disease genes in human Mol Biol Evol 2010 27 934 941 10.1093/molbev/msp297 19955474

[B13] Podder S Ghosh TC Insights into the molecular correlates modulating functional compensation between monogenic and polygenic disease gene duplicates in human Genomics 2011 97 200 204 10.1016/j.ygeno.2011.01.004 21281709

[B14] Sabeti PC Varilly P Fry B Lohmueller J Hostetter E Cotsapas C Xie X Byrne EH McCarroll SA Gaudet R Genome-wide detection and characterization of positive selection in human populations Nature 2007 449 913 918 10.1038/nature06250 17943131 PMC2687721

[B15] Chen FC Wang SS Chen CJ Li WH Chuang TJ Alternatively and constitutively spliced exons are subject to different evolutionary forces Mol Biol Evol 2006 23 675 682 16368777 10.1093/molbev/msj081

[B16] Stephan W Langley CH Molecular genetic variation in the centromeric region of the X chromosome in three Drosophila ananassae populations. I. Contrasts between the vermilion and forked loci Genetics 1989 121 89 99 2563714 10.1093/genetics/121.1.89 PMC1203608

[B17] Begun DJ Aquadro CF Molecular population genetics of the distal portion of the X chromosome in Drosophila: evidence for genetic hitchhiking of the yellow-achaete region Genetics 1991 129 1147 1158 1664405 10.1093/genetics/129.4.1147 PMC1204778

[B18] Nachman MW Bauer VL Crowell SL Aquadro CF DNA variability and recombination rates at X-linked loci in humans Genetics 1998 150 1133 1141 9799265 10.1093/genetics/150.3.1133 PMC1460397

[B19] Dvorák J Luo MC Yang ZL Restriction fragment length polymorphism and divergence in the genomic regions of high and low recombination in self-fertilizing and cross-fertilizing aegilops species Genetics 1998 148 423 434 9475752 10.1093/genetics/148.1.423 PMC1459766

[B20] Hill WG Robertson A The effect of linkage on limits to artificial selection Genet Res 1966 8 269 294 10.1017/S0016672300010156 5980116

[B21] Connallon T Knowles LL Recombination rate and protein evolution in yeast BMC Evol Biol 2007 7 235 10.1186/1471-2148-7-235 18042299 PMC2211315

[B22] Pál C Papp B Hurst LD Does the recombination rate affect the efficiency of purifying selection? The yeast genome provides a partial answer Mol Biol Evol 2001 18 2323 2326 10.1093/oxfordjournals.molbev.a003779 11719582

[B23] Zhang L Lu HH Chung WY Yang J Li WH Patterns of segmental duplication in the human genome Mol Biol Evol 2005 22 135 141 15371527 10.1093/molbev/msh262

[B24] Sen K Podder S Ghosh TC Insights into the genomic features and evolutionary impact of the genes configuring duplicated pseudogenes in human FEBS Lett 2010 584 4015 4018 10.1016/j.febslet.2010.08.012 20708614

[B25] Jordan IK Wolf YI Koonin EV Duplicated genes evolve slower than singletons despite the initial rate increase BMC Evol Biol 2004 4 22 10.1186/1471-2148-4-22 15238160 PMC481058

[B26] Hastings PJ Lupski JR Rosenberg SM Ira G Mechanisms of change in gene copy number Nat Rev Genet 2009 10 551 564 19597530 10.1038/nrg2593 PMC2864001

[B27] Nguyen DQ Webber C Hehir-Kwa J Pfundt R Veltman J Ponting CP Reduced purifying selection prevails over positive selection in human copy number variant evolution Genome Res 2008 18 1711 1723 10.1101/gr.077289.108 18687881 PMC2577867

[B28] Nguyen DQ Webber C Ponting CP Bias of selection on human copy-number variants PLoS Genet 2006 2 e20 10.1371/journal.pgen.0020020 16482228 PMC1366494

[B29] Modrek B Lee C A genomic view of alternative splicing Nat Genet 2002 30 13 19 10.1038/ng0102-13 11753382

[B30] Ramensky VE Nurtdinov RN Neverov AD Mironov AA Gelfand MS Positive selection in alternatively spliced exons of human genes Am J Hum Genet 2008 83 94 98 10.1016/j.ajhg.2008.05.017 18571144 PMC2443848

[B31] Chen FC Wang SS Chen CJ Li WH Chuang TJ Alternatively and constitutively spliced exons are subject to different evolutionary forces Mol Biol Evol 2006 23 675 682 16368777 10.1093/molbev/msj081

[B32] Lynch KW Consequences of regulated pre-mRNA splicing in the immune system Nat Rev Immunol 2004 4 931 940 10.1038/nri1497 15573128

[B33] Zhang H Wang L Song L Zhao J Qiu L Gao Y Song X Li L Zhang Y Zhang L The genomic structure, alternative splicing and immune response of Chlamys farreri thioester-containing protein Dev Comp Immunol 2009 33 1070 1076 10.1016/j.dci.2009.05.007 19467260

[B34] Lewis BP Green RE Brenner SE Evidence for the widespread coupling of alternative splicing and nonsense-mediated mRNA decay in humans Proc Natl Acad Sci USA 2003 100 189 192 10.1073/pnas.0136770100 12502788 PMC140922

[B35] McGlincy NJ Smith CW Alternative splicing resulting in nonsense-mediated mRNA decay: what is the meaning of nonsense? Trends Biochem Sci 2008 33 385 393 10.1016/j.tibs.2008.06.001 18621535

[B36] Edwards YJ Lobley AE Pentony MM Jones DT Insights into the regulation of intrinsically disordered proteins in the human proteome by analyzing sequence and gene expression data Genome Biol 2009 10 R50 10.1186/gb-2009-10-5-r50 19432952 PMC2718516

[B37] Romero PR Zaidi S Fang YY Uversky VN Radivojac P Oldfield CJ Cortese MS Sickmeier M LeGall T Obradovic Z Dunker AK Alternative splicing in concert with protein intrinsic disorder enables increased functional diversity in multicellular organisms Proc Natl Acad Sci USA 2006 103 8390 8395 10.1073/pnas.0507916103 16717195 PMC1482503

[B38] Kovacs E Tompa P Liliom K Kalmar L Dual coding in alternative reading frames correlates with intrinsic protein disorder Proc Natl Acad Sci USA 2010 107 5429 5434 10.1073/pnas.0907841107 20212158 PMC2851785

[B39] Uversky VN Oldfield CJ Dunker AK Showing your ID: intrinsic disorder as an ID for recognition, regulation and cell signaling J Mol Recognit 2005 18 343 384 10.1002/jmr.747 16094605

[B40] Brown CJ Takayama S Campen AM Vise P Marshall TW Oldfield CJ Williams CJ Dunker AK Evolutionary rate heterogeneity in proteins with long disordered regions J Mol Evol 2002 55 104 110 10.1007/s00239-001-2309-6 12165847

[B41] Montgomery SB Sammeth M Gutierrez-Arcelus M Lach RP Ingle C Nisbett J Guigo R Dermitzakis ET Transcriptome genetics using second generation sequencing in a Caucasian population Nature 2010 464 773 777 10.1038/nature08903 20220756 PMC3836232

[B42] Krawczak M Reiss J Cooper DN The mutational spectrum of single base-pair substitutions in mRNA splice junctions of human genes: causes and consequences Hum Genet 1992 90 41 54 1427786 10.1007/BF00210743

[B43] Krawczak M Thomas NS Hundrieser B Mort M Wittig M Hampe J Cooper DN Single base-pair substitutions in exon-intron junctions of human genes: nature, distribution, and consequences for mRNA splicing Hum Mutat 2007 28 150 158 10.1002/humu.20400 17001642

[B44] Walsh CT Posttranslational modification of proteins: expanding nature's inventory 2006 Englewood, CO: Roberts and Company Publishers

[B45] Cloos PA Christgau S Post-translational modifications of proteins: implications for aging, antigen recognition, and autoimmunity Biogerontology 2004 5 139 158 15190184 10.1023/B:BGEN.0000031152.31352.8b

[B46] Gray VE Kumar S Rampant purifying selection conserves positions with posttranslational modifications in human proteins Mol Biol Evol 2011 28 1565 1568 10.1093/molbev/msr013 21273632 PMC3107664

[B47] Ponder JW Richards FM Tertiary templates for proteins. Use of packing criteria in the enumeration of allowed sequences for different structural classes J Mol Biol 1987 193 775 791 10.1016/0022-2836(87)90358-5 2441069

[B48] Goldman N Thorne JL Jones DT Assessing the impact of secondary structure and solvent accessibility on protein evolution Genetics 1998 149 445 458 9584116 10.1093/genetics/149.1.445 PMC1460119

[B49] Sunyaev S Ramensky V Koch I Lathe W Kondrashov AS Bork P Prediction of deleterious human alleles Hum Mol Genet 2001 10 591 597 10.1093/hmg/10.6.591 11230178

[B50] Chakraborty S Kahali B Ghosh TC Protein complex forming ability is favored over the features of interacting partners in determining the evolutionary rates of proteins in the yeast protein-protein interaction networks BMC Syst Biol 2010 4 155 10.1186/1752-0509-4-155 21073713 PMC2998497

[B51] Smith NG Eyre-Walker A Human disease genes: patterns and predictions Gene 2003 318 169 175 14585509 10.1016/s0378-1119(03)00772-8

[B52] López-Bigas N Ouzounis CA Genome-wide identification of genes likely to be involved in human genetic disease Nucleic Acids Res 2004 32 3108 3114 10.1093/nar/gkh605 15181176 PMC434425

[B53] Greco D Somervuo P Di Lieto A Raitila T Nitsch L Castrén E Auvinen P Physiology, pathology and relatedness of human tissues from gene expression meta-analysis PLoS One 2008 3 e1880 10.1371/journal.pone.0001880 18382664 PMC2268968

[B54] Conrad B Antonarakis SE Gene duplication: a drive for phenotypic diversity and cause of human disease Annu Rev Genomics Hum Genet 2007 8 17 35 10.1146/annurev.genom.8.021307.110233 17386002

[B55] Su Z Wang J Yu J Huang X Gu X Evolution of alternative splicing after gene duplication Genome Res 2006 16 182 189 16365379 10.1101/gr.4197006 PMC1361713

[B56] Cuccurese M Russo G Russo A Pietropaolo C Alternative splicing and nonsense-mediated mRNA decay regulate mammalian ribosomal gene expression Nucleic Acids Res 2005 33 5965 5977 10.1093/nar/gki905 16254077 PMC1270949

[B57] Khadra A Santamaria P Edelstein-Keshet L The pathogenicity of self-antigen decreases at high levels of autoantigenicity: a computational approach Int Immunol 2010 22 571 582 10.1093/intimm/dxq041 20497954 PMC2892361

[B58] Becker KG Barnes KC Bright TJ Wang SA The genetic association database Nat Genet 2004 36 431 432 10.1038/ng0504-431 15118671

[B59] Collison LW Chaturvedi V Henderson AL Giacomin PR Guy C Bankoti J Finkelstein D Forbes K Workman CJ Brown SA IL-35-mediated induction of a potent regulatory T cell population Nat Immunol 2010 11 1093 1101 10.1038/ni.1952 20953201 PMC3008395

[B60] Flicek P Amode MR Barrell D Beal K Brent S Chen Y Clapham P Coates G Fairley S Fitzgerald S Ensembl 2011 Nucleic Acids Res 2011 39 D800 806 10.1093/nar/gkq1064 21045057 PMC3013672

[B61] Su AI Wiltshire T Batalov S Lapp H Ching KA Block D Zhang J Soden R Hayakawa M Kreiman G A gene atlas of the mouse and human protein-encoding transcriptomes Proc Natl Acad Sci USA 2004 101 6062 6067 10.1073/pnas.0400782101 15075390 PMC395923

[B62] Podder S Mukhopadhyay P Ghosh TC Multifunctionality dominantly determines the rate of human housekeeping and tissue specific interacting protein evolution Gene 2009 439 11 16 10.1016/j.gene.2009.03.005 19306918

[B63] Jegga AG Gowrisankar S Chen J Aronow BJ PolyDoms: a whole genome database for the identification of non-synonymous coding SNPs with the potential to impact disease Nucleic Acids Res 2007 35 D700 706 10.1093/nar/gkl826 17142238 PMC1669724

[B64] Zhang J Feuk L Duggan GE Khaja R Scherer SW Development of bioinformatics resources for display and analysis of copy number and other structural variants in the human genome Cytogenet Genome Res 2006 115 205 214 10.1159/000095916 17124402

[B65] Thorisson GA Smith AV Krishnan L Stein LD The International HapMap Project Web site Genome Res 2005 15 1592 1593 10.1101/gr.4413105 16251469 PMC1310647

[B66] Kato M Miya F Kanemura Y Tanaka T Nakamura Y Tsunoda T Recombination rates of genes expressed in human tissues Hum Mol Genet 2008 17 577 586 18000027 10.1093/hmg/ddm332

[B67] Lee C Atanelov L Modrek B Xing Y ASAP: the Alternative Splicing Annotation Project Nucleic Acids Res 2003 31 101 105 10.1093/nar/gkg029 12519958 PMC165476

[B68] Yang JO Kim WY Bhak J ssSNPTarget: genome-wide splice-site Single Nucleotide Polymorphism database Hum Mutat 2009 30 E1010 1020 10.1002/humu.21128 19760752

[B69] Shionyu M Yamaguchi A Shinoda K Takahashi K Go M AS-ALPS: a database for analyzing the effects of alternative splicing on protein structure, interaction and network in human and mouse Nucleic Acids Res 2009 37 D305 309 10.1093/nar/gkn869 19015123 PMC2686549

[B70] Prilusky J Felder CE Zeev-Ben-Mordehai T Rydberg EH Man O Beckmann JS Silman I Sussman JL FoldIndex: a simple tool to predict whether a given protein sequence is intrinsically unfolded Bioinformatics 2005 21 3435 3438 10.1093/bioinformatics/bti537 15955783

[B71] Uversky VN Gillespie JR Fink AL Why are "natively unfolded" proteins unstructured under physiologic conditions? Proteins 2000 41 415 427 10.1002/1097-0134(20001115)41:3<415::AID-PROT130>3.0.CO;2-7 11025552

[B72] Blom N Gammeltoft S Brunak S Sequence and structure-based prediction of eukaryotic protein phosphorylation sites J Mol Biol 1999 294 1351 1362 10.1006/jmbi.1999.3310 10600390

[B73] Gasteiger E Gattiker A Hoogland C Ivanyi I Appel RD Bairoch A ExPASy: The proteomics server for in-depth protein knowledge and analysis Nucleic Acids Res 2003 31 3784 3788 10.1093/nar/gkg563 12824418 PMC168970

[B74] Ahmad S Gromiha MM Sarai A RVP-net: online prediction of real valued accessible surface area of proteins from single sequences Bioinformatics 2003 19 1849 1851 10.1093/bioinformatics/btg249 14512359

[B75] D'Onofrio G Jabbari K Musto H Bernardi G The correlation of protein hydropathy with the base composition of coding sequences Gene 1999 238 3 14 10.1016/S0378-1119(99)00257-7 10570978

